# Factors associated with mother's healthcare-seeking behavior for symptoms of acute respiratory infection in under-five children in sub-Saharan Africa: a multilevel robust Poisson regression modelling

**DOI:** 10.1186/s12913-023-10065-x

**Published:** 2023-10-05

**Authors:** Getayeneh Antehunegn Tesema, Beminate Lemma Seifu

**Affiliations:** 1 Department of Epidemiology and Biostatistics, Institute of Public Health, College of Medicine and Health Sciences and Comprehensive Specialized Hospital, University of Gondar, Gondar, Ethiopia; 2 Department of Public Health, College of Medicine and Health Sciences, Samara University, Samara, Ethiopia

**Keywords:** Under-five children, Multilevel robust Poisson regression, SSA, Mothers healthcare-seeking behavior

## Abstract

**Background:**

Timely and appropriate treatment for childhood illness saves the lives of millions of children. In low-middle-income countries such as sub-Saharan Africa (SSA), poor healthcare-seeking behavior for childhood illnesses is identified as a major contributor to the increased risk of child morbidity and mortality. However, studies are limited on Factors associated with mother's healthcare-seeking behavior for symptoms of acute respiratory infection in under-five children in sub-Saharan Africa.

**Objective:**

To examine factors associated with a mother's healthcare-seeking behavior for symptoms of acute respiratory infection in under-five children in sub-Saharan Africa.

**Methods:**

A secondary data analysis was conducted based on the latest Demographic and Health Survey (DHS) data of 36 sub-Saharan African countries. A total weighted sample of 16,925 mothers who had under-five children with acute respiratory infection symptoms was considered. The Intraclass Correlation Coefficient (ICC), Median Odds Ratio (MOR), and Likelihood Ratio (LR) tests were done to assess the presence of clustering. Model comparison was made based on deviance (-2LLR) value. Variables with a *p*-value < 0.2 in the bivariable multilevel robust Poisson analysis were considered for the multivariable analysis. In the multivariable multilevel robust Poisson regression analysis, the Adjusted Prevalence Ratio (APR) with the 95% Confidence Interval (CI) was reported to declare the statistical significance and strength of the association.

**Results:**

The prevalence of mother's healthcare-seeking behavior for symptoms of acute respiratory infection in under-five children in SSA was 64.9% (95% CI: 64.2%, 65.7%). In the multivariable analysis; mothers who attained primary education (APR = 1.11, 95% CI: 1.08, 1.15), secondary education (APR = 1.13, 95% CI: 1.09, 1.18), and higher education (APR = 1.19, 95% CI: 1.11, 1.27), belonged to the richest household (APR = 1.07: 95% CI: 1.02, 1.12), had media exposure (APR = 1.11, 95% CI: 1.08, 1.15), currently working (APR = 1.08, 95% CI: 1.06, 1.11), had ANC use (APR = 1.25: 95% CI: 1.17, 1.35), health facility delivery (APR = 1.10, 95% CI: 1.07, 1.14), belonged to West Africa (APR = 1.04, 95% CI: 1.01, 1.08) and being in the community with high media exposure (APR = 1.04, 95% CI: 1.02, 1,07) were significantly associated with higher prevalence of mother's healthcare-seeking behavior for symptoms of acute respiratory infection in under-five children. On the other hand, distance to a health facility (APR = 0.87, 95% CI: 0.84, 0.91), and being in central Africa (APR = 0.87, 95% CI: 0.84, 0.91) were significantly associated with a lower prevalence of mother's healthcare-seeking behavior for symptoms of acute respiratory infection in under-five children.

**Conclusion:**

Mother's healthcare-seeking behavior for symptoms of acute respiratory infection in under-five children. It was influenced by maternal education, maternal working status, media exposure, household wealth status, distance to the health facility, and maternal health care service use. Any interventions aiming at improving maternal education, maternal healthcare services, and media access are critical in improving mothers' healthcare-seeking behavior for symptoms of acute respiratory infection in under-five children, hence lowering the prevalence of ARI-related death and morbidity.

## Background

Globally, acute Respiratory Infections (ARIs) are the leading causes of under-five morbidity and mortality [1, 2]. They account for 6% of the worldwide disease burden [3–5]. While sub-Saharan Africa (SSA) has made significant progress in lowering under-five mortality, the progress is far below the expected [6–8]. ARIs are caused by a wide range of pathogens such as *Streptococcus pneumonia, Haemophilus influenzae, Staphylococcus aureus*, etc. [9].

Effective antibiotic treatment for ARIs is available, and timely treatment can prevent the majority of ARI-related deaths [10, 11]. Effective treatment of acute respiratory infections in children under the age of five requires early diagnosis, timely medical attention seeking, and administration of the necessary drugs [12]. However, less than one-third of ARI cases in low-income countries [13], and only 40% of children under the age of five in SSA with ARI symptoms obtained medical treatment [14].

One of the key components of the Integrated Management of Childhood Illness Strategy (IMCIS) is the World Health Organization's (WHO) effort to improve family and community healthcare practices towards disease detection and care-seeking behaviors [15, 16]. However, IMCIS implemented in Low and Middle-income Countries (LMIC) specifically in SSA has not achieved its target [17]. Mothers or caregivers play a crucial role in recognizing the symptoms of acute respiratory infection in under-five children [18, 19]. Previous studies conducted on mother's healthcare-seeking behavior for symptoms of acute respiratory infection in under-five children showed that maternal education [20, 21], household wealth status [19, 22], distance to a health facility [23, 24], residence [20, 25], childhood nutritional status [19, 26], maternal occupation [22, 27], husband education [28], perceived severity [20, 21, 29], previous history of under-five death [20], media exposure [14], ANC visit [30], and place of delivery [31, 32] were found to be significant predictors.

Despite the ongoing advancement of medicine, ARIs continue to impose a significant burden on under-five morbidity and mortality in sub-Saharan African countries. This is closely linked to failure to seek health care and delay in seeking timely and appropriate care. In addition, ARIs continue to be one of the major health issues in SSA, and as far as we are concerned a study on healthcare-seeking behavior for symptoms of ARIs among under-five children and associated factors in SSA using a multilevel robust Poisson regression model has not yet been done. Even though the mother's healthcare-seeking behavior was not a rare occurrence, prior studies in different Sub-Saharan African countries reported odds ratios to quantify the association [33–35]. When the prevalence exceeds 10%, using a multilevel Poisson regression model with robust error variance in cross-sectional studies prevents overestimation of the association between outcome and explanatory variables [36]. As a result, we examined mothers' healthcare-seeking behavior in SSA for under-five children with ARI symptoms.

## Methods

### Study design and settings

A secondary data analysis was conducted based on the most recent Demographic and Health Surveys (DHSs) of 36 sub-Saharan African countries conducted from 2005 to 2019. DHS is a community-based cross-sectional study conducted in five-year intervals to generate basic health and health-related indicators of the population.

### Study population and sampling

The study population was the mothers who had under-five children with ARI symptoms. Two-stage stratified cluster sampling technique was employed using Enumeration Areas (EAs) as primary sampling units and households as secondary sampling units [37]. The Kids Record dataset (KR file) was used for this study after we obtained an authorization letter from the measure DHS program for data access. A total of 16,925 weighted samples were considered for this study. The detailed methodology is available in the following references [38, 39].

### Measurement of variables

#### Dependent variable

Mother's healthcare-seeking behavior for symptoms of acute respiratory infection in under-five children was the dependent variable. The presence of ARIs is defined as children having a history of cough accompanied by short, rapid breathing or difficulty breathing and fever within two weeks preceding the survey. In DHS, mothers of under-five children were asked whether their children had a history of cough within two weeks preceding the survey. For children who had a cough, the mother was asked whether the child's cough was accompanied by short, rapid breathing or difficulty breathing and fever within two weeks preceding the survey. It was obtained from the DHS question "Did he/she breathe faster than usual with short, rapid breaths or have difficulty breathing in the 2 weeks preceding the survey?”. Then classified as "yes" if a child meets all the aforementioned conditions and "no" if a child does not [40]. This study was limited to mothers who had children aged 0–59 months with symptoms of ARIs within two weeks preceding the survey. Following that, it was categorized as "Yes" if they sought medical attention for their ARI symptoms, and "No" if they did not.

### Independent variables

Maternal age, maternal educational status, household wealth status, media exposure, sex of a child, birth size, place of delivery, had ANC visit, maternal working status, stunting, wasting, underweight, marital status, and husband education were level one (individual level) variables. On the other hand, residence, sub-Saharan African region, distance to the health facility, community media exposure, community maternal education, and community poverty were level two (community level) variables.

To assess a child's nutritional status DHS used anthropometric measures (height, age, and weight), height for age measures stunting, weight for height measured wasting, and weight for age measured for underweight. Compared with the reference population of children, the children < -3 standard deviations were severe malnutrition and between -3 and -2 standard deviations were moderate.

Media exposure was calculated by aggregating three variables such as watching television, listening to the radio, and reading newspapers. Then, categorized as having media exposure if a mother has been exposed to at least one of the three and not if she had no exposure to any of the media sources.

In DHS data, there was no variable collected at the community level except residence, and distance to the health facility. Therefore, we generated community media exposure by aggregating listening to the radio, watching television, and reading newspapers at the cluster level. These were categorized as higher community media exposure and lower media exposure based on the national median value of media exposure since it was not normally distributed. Community maternal education and community poverty were generated by aggregating maternal education and wealth status at the cluster/enumeration area levels. Then categorized as higher community maternal education and poverty based on the national median value of maternal education and poverty since they were not normally distributed.

### Data management and analysis

STATA version 17 and R version 4.1.3 statistical software were used for data management and analysis. All the results were based on the weighted data. DHS is a cross-sectional study, and the prevalence of mothers seeking healthcare for ARI symptoms in children under the age of five was 64.9%, which was larger than 10%. In this scenario, reporting the odds ratio could overestimate the relationship between the independent variables and the mother's healthcare-seeking behavior for symptoms of ARIs in children under the age of five. Therefore, the prevalence ratio is the best measure of association for the current study. To obtain the prevalence ratio, we have fitted a multilevel Poisson regression model with robust variance.

We preferred this model for three reasons. Firstly, when the magnitude of the outcome variable is common, the odds ratio obtained using the binary logistic regression overestimates the strength of the relationship. Secondly, because the DHS data is hierarchical, mothers were nested within cluster/EA. Thirdly, the multilevel robust Poisson regression model outperformed the multilevel log-binomial regression model in terms of convergence. Therefore, this model accounts for data dependencies as well as the problem of overestimation.

Likelihood Ratio (LR) test, Intra-class Correlation Coefficient (ICC), and Median Odds Ratio (MOR) were computed to measure the variation between clusters. The ICC quantifies the degree of heterogeneity between clusters (the proportion of the total observed individual variation in mothers' healthcare-seeking behavior for symptoms of ARIs among children under age five that is attributable to cluster variations) [41].

$$ICC= {\sigma }^{2 }+\left({\sigma }^{2}+{}^{{\pi }^{2}}\!\left/ \!{}_{3}\right.\right)$$

π^2^/3 is the individual-level variance which is approximated to 3.29, and ${\sigma }^{2}$ represents the community-level variance

But MOR is quantifying the variation or heterogeneity in outcomes between clusters. It is defined as the median value of the odds ratio between the cluster more likely to seek health care for symptoms of ARIs of children under age five and the cluster at lower risk when randomly picking out two clusters (EAs) [42].

$$\mathrm{MOR}\hspace{0.17em}=\hspace{0.17em}\mathrm{exp\;}(\sqrt{2*\partial 2*0.6745}) \sim \mathrm{ MOR}\hspace{0.17em}=\hspace{0.17em}\mathrm{exp\;}(0.95*\partial )$$

We have fitted four models separately. Model 1 (null model) was fitted without an independent variable to estimate the cluster-level variation of the mother's healthcare-seeking behavior in SSA. Model 2 and Model 3 were adjusted for individual-level variables and community-level variables, respectively. Model 4 was the final model adjusted for individual and community-level variables simultaneously. Variables with a *p*-value < 0.2 in the bi-variable multilevel Poisson regression analysis were considered for the multivariable analysis. Deviance (-2Log-likelihood Ratio (-2LLR)) was used to compare models, and a model with the lowest deviance was considered the best-fit model. Finally, the Adjusted Prevalence Ratio (APR) with its 95% confidence interval (CI) was reported to declare the statistical significance and strength of the association.

### Ethical consideration

Permission to get access to the data was obtained from the measure DHS program online request from http://www.dhsprogram.com.website and the data used were publicly available with no personal identifier. The data used for this study were publicly available with no personal identifier. For the details see https://dhsprogram.com/methodology/Protecting-the-Privacy-of-DHS-SurveyRespondents.cfm.

## Results

### Descriptive characteristics of mothers of children under age five with ARI symptoms

A total of 16,925 mothers of children under the age of five with symptoms of ARI were included in this study. More than half (52.1%) of the children were males and nearly one-third (30.9%) of mothers were aged 15–24 years. About 4,224 (25%) and 2,456 (14.5%) of the children belonged to the poorest and richest households, respectively. The majority (72.3%) of children lived in rural areas, and 42.4% were in East African countries. About 43.4%, 36.5%, and 45.6% of children under age five were severely wasted, severely stunted, and severely underweight, respectively (Table 1).

**Table 1:** Demographic, socio-economic, and health-related characteristics of mothers of under-five children with symptoms of ARIs

Variables	Frequency (*n* =	Percentage (%)
**Residence**		
Urban	4695	27.7
Rural	12,230	72.3
**Maternal age**		
15–24	5222	30.9
25–34	8105	47.9
≥ 35	3598	21.2
**Maternal education status**		
No	5924	35.0
Primary	6836	40.4
Secondary	3737	22.1
Higher	428	2.5
**Household wealth status**		
Poorest	4224	25.0
Poorer	3798	22.4
Middle	3347	19.8
Richer	3100	18.3
Richest	2456	14.5
**Media exposure**		
No	5718	33.8
Yes	11,207	66.2
**Sex of child**		
Male	8811	52.1
Female	8114	47.9
**Birth size**		
Average	6611	39.1
Small	4145	24.5
Large	6169	36.4
**Place of delivery**		
Home	5969	35.3
Health facility	10,956	64.7
**Had ANC visit**		
No	1180	7.0
Yes	15,745	93.0
**Distance to a health facility**		
Not a big problem	10,166	60.1
A big problem	6759	39.9
**Maternal working status**		
Not working	5567	34.6
Working	10,517	65.4
**Stunting**		
Normal	6642	39.2
Moderate	1899	11.2
Severe	6169	36.5
**Wasting**		
Normal	9018	53.3
Moderate	555	3.3
Severe	7352	43.4
**Underweight**		
Normal	7992	47.2
Moderate	1216	7.2
Severe	7717	45.6
**Marital status**		
Single	1034	6.1
Married	14,662	86.6
Widowed	220	1.3
Divorced	1010	6.0
**Husband’s education status (*n* = 14,816)**		
No	4475	31.6
Primary	4852	34.2
Secondary	4098	28.9
Higher	761	5.4
**sub-Saharan Africa region**		
East Africa	7179	42.4
Central Africa	4245	25.1
Southern Africa	714	4.2
West Africa	4787	28.3
**Community media exposure**		
Low	9113	53.8
High	7812	46.7
**Community maternal education**		
Low	13,931	82.3
High	2994	17.7
**Community poverty**		
Low	9404	55.6
High	7521	44.4

### Prevalence of mothers’ healthcare-seeking behavior for symptoms of ARIs among children under age five in SSA

**Fig. 1:**
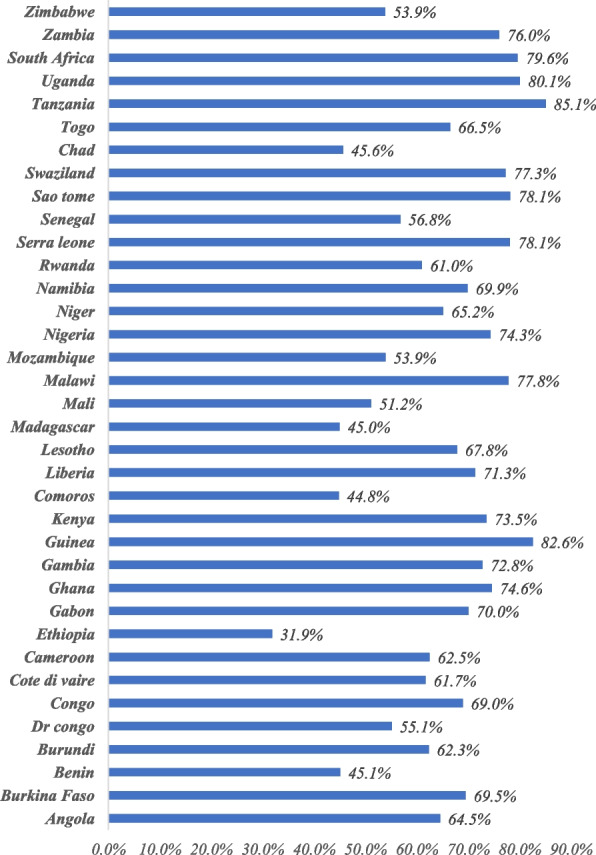
The percentage of mothers’ healthcare-seeking behaviour across countries

The prevalence of mothers’ healthcare-seeking behavior for symptoms of ARIs among children under age five in SSA was 64.9% (95% CI: 64.2%, 65.7%), it ranged from 31.9% in Ethiopia to 85.1% in Tanzania (Fig. 1). The prevalence of mothers' healthcare-seeking behavior for symptoms of ARIs among children had significant differences by residence, maternal age, maternal education, mothers working status, SSA region, community media exposure, media exposure, community maternal education, wealth status, community poverty, distance to health facility, place of delivery, ANC visit, stunting status, wasting status and underweight status (*p* < 0.05) (Table 2).

**Table 2:** Distribution of study participants characteristics by their healthcare seeking behaviour for symptoms of acute respiratory infections in under-five children in SSA

Variables	Did not seek medical care	Sought medical care	*P*-value
**Residence**			
Urban	1322 (28.15)	3374 (71.85)	0.00001
Rural	4612 (37.71)	7618 (62.29)	
**Maternal age**			
15–24	1755 (33.61)	3466 (66.39)	0.018
25–34	2901 (35.79)	5204 (64.21)	
≥ 35	1278 (35.51)	2321 (64.49)	
**Maternal education status**			
No	2591 (43.74)	3333 (56.26)	0.00001
Primary	2284 (33.41)	4552 (66.59)	
Secondary	967 (25.87)	2770 (74.13)	
Higher	91 (21.33)	337 (78.67)	
**Household wealth status**			
Poorest	1739 (41.18)	2484 (58.82)	0.00001
Poorer	1387 (36.52)	2411 (63.48)	
Middle	1202 (35.91)	2145 (64.09)	
Richer	1011 (32.60)	2089 (67.40)	
Richest	595 (24.21)	1861 (75.79)	
**Media exposure**			
No	2576 (45.05)	3142 (54.95)	0.0001
Yes	3358 (29.96)	7849 (70.04)	
**Sex of child**			
Male	3010 (34.16)	5801 (65.84)	0.144
Female	2924 (36.03)	5190 (63.97)	
**Birth size**			
Average	2215 (33.49)	4397 (66.51)	0.238
Small	1526 (36.81)	2619 (63.19)	
Large	2194 (35.56)	3975 (64.44)	
**Place of delivery**			
Home	2649 (44.37)	3320 (55.63)	0.0001
Health facility	3285 (29.98)	7671 (70.02)	
**Had ANC visit**			
No	680 (57.64)	500 (42.36)	0.0001
Yes	5253 (33.37)	10,492 (66.63)	
**Distance to a health facility**			
Not a big problem	3310 (32.56)	6857 (67.44)	0.0001
A big problem	2623 (38.82)	4135 (61.18)	
**Maternal working status**			
Not working	2479 (38.69)	3929 (61.31)	0.0001
Working	3454 (32.85)	7063 (67.15)	
**Stunting**			
Normal	2383 (35.87)	4259 (64.13)	0.044
Moderate	719 (37.88)	1180 (62.12)	
Severe	2831 (33.77)	5553 (66.23)	
**Wasting**			
Normal	3283 (36.40)	5735 (63.60)	0.001
Moderate	222 (40.08)	333 (59.92)	
Severe	2429 (33.03)	4924 (66.97)	
**Underweight**			
Normal	2833 (35.45)	5159 (64.55)	0.0001
Moderate	500 (41.13)	716 (58.87)	
Severe	2600 (33.69)	5117 (66.31)	
**Marital status**			
Single	291 (28.13)	743 (71.87)	0.0001
Married	5241 (35.75)	9421 (64.25)	
Widowed	73 (33.51)	146 (66.49)	
Divorced	328 (32.51)	682 (67.49)	
**Husband’s education status (*n* = 14,816)**			
No	1934 (43.22)	2541 (56.78)	0.0001
Primary	1725 (35.56)	3126 (64.44)	
Secondary	1325 (32.32)	2774 (67.68)	
Higher	161 (21.20)	600 (78.80)	
**sub-Saharan Africa region**			
East Africa	2368 (32.99)	4811 (67.01)	0.0001
Central Africa	1799 (42.38)	2446 (57.62)	
West Africa	1575 (32.89)	3212 (67.11)	
Southern Africa	192 (26.85)	522 (73.15)	
**Community media exposure**			
Low	3546 (38.91)	5567 (61.09)	0.0001
High	2388 (30.57)	5424 (69.43)	
**Community maternal education**			
Low	5060 (36.32)	8871 (63.68)	0.0001
High	874 (29.19)	2120 (70.81)	
**Community poverty**			
Low	3176 (33.78)	6228 (66.22)	0.013
High	2757 (36.66)	4764 (63.34)	

### Associated factors of mother's healthcare-seeking behavior for symptoms of acute respiratory infection in under-five children in SSA

In the null model, there was statistically significant variability in the odds of the mother's healthcare-seeking behavior for symptoms of acute respiratory infection in under-five children between clusters (the LR test was statistically significant (*p* = 0.0001)). Though the ICC value in the null model was 3.4%, the MOR revealed that if we randomly select two mothers from different clusters and transfer women from the cluster with a lower likelihood of health care seeking behavior to cluster with higher healthcare-seeking behavior, she could have 1.42 times higher prevalence of seeking health care for symptoms of ARI. Therefore, a multilevel robust Poisson regression model was fitted, and the final model was the best-fitted model since it has the lowest deviance value (deviance = 31,006,76).

In the final multivariable multilevel robust Poisson regression model; maternal educational status, household wealth status, media exposure, mother working status, ANC visits, and place of delivery were among the individual factors associated with mothers' healthcare-seeking behavior for symptoms of ARIs among children under age five. Sub-Saharan African region, distance to the health facility, and community media exposure were among the community-level factors associated with mothers' healthcare-seeking behavior for symptoms of ARIs in children under age five.

Mothers' who attained primary, secondary, and higher education had 1.11 times (APR = 1.11, 95% CI: 1.08, 1.15), 1.13 times (APR = 1.13, 95% CI: 1.09, 1.18), and 1.19 times (APR = 1.19, 95% CI: 1.11, 1,27) higher prevalence of seeking health care for symptoms of ARIs of children under age five than mothers who had no formal education, respectively. The prevalence of seeking health care for symptoms of children under age five among mothers belonging to the richest household was 1.07 times (APR = 1.07, 95% CI: 1.02, 1.12) higher than mothers belonging to the poorest household. Mothers who had media exposure had 1.11 times (APR = 1.11, 95% CI: 1.08, 1.15) higher prevalence of seeking health care for symptoms of ARIs than those who had no exposure to media. Compared to mothers who had no work, mothers who were working had 1.08 times (APR = 1.08, 95% CI: 1.06, 1.11) higher prevalence of seeking health care for symptoms of ARIs in children under five age. Mothers who had ANC visits for the index pregnancy had 1.25 times (APR = 1.25, 95% CI: 1.17, 1.35) higher prevalence of seeking health care for symptoms of ARI than those who had no ANC visits. Similarly, the prevalence of seeking health care for symptoms of ARI in children under age five among mothers who gave birth at a health facility was 1.10 times (APR = 1.10, 95% CI: 1.07, 1.14) higher compared to mothers who gave birth at home.

The prevalence of seeking healthcare for symptoms of ARIs among children under the age of five was decreased by 13% (APR = 0.87, 95% CI: 0.84, 0.91) among mothers in East Africa. Whereas mothers in West Africa had 1.04 times (APR = 1.04, 95% APR = 1.01, 1,08) higher prevalence of seeking healthcare for symptoms of ARIs than mothers in East Africa. Regarding distance to the health facility, mothers who had a big problem with the health facility had 0.95 times (APR = 0.95, 95% CI: 0.93, 0.98) decreased prevalence of seeking healthcare for symptoms of ARIs. Being from communities with a high level of media exposure had 1.04 times (APR = 1.04, 95% CI: 1.02, 1.07) higher prevalence of seeking healthcare for children under age five compared to mothers in the community with a low level of media exposure (Table 3).

**Table 3:** Multilevel robust Poisson regression analysis of factors associated with mother's healthcare-seeking behaviour for symptoms of ARIs among children under age five in sub-Saharan Africa

Variables	Null model	Model I (Individual-level variables)	Model II (Community level characteristics)	Model III (Both individual and community level variables)
		**PR with 95% CI**	**PR with 95% CI**	**PR with 95% CI**
**Maternal age (in years)**				
15–24		1		1
25–34		0.97 (0.95, 1.00)		0.97 (0.95, 1.00)
≥ 35		0.99 (0.95, 1.02)		0.99 (0.95, 1.02)
**Maternal education status**				
No		1		1
Primary		1.09 (1.06, 1.13)		1.11 (1.08, 1.15)^**^
Secondary		1.10 (1.06, 1.14)		1.13 (1.09, 1.18)^**^
Higher		1.17 (1.10,1.25)		1.19 (1.11, 1.27)^**^
**Household wealth status**				
Poorest		1		1
Poorer		1.03 (0.99, 1.07)		1.02 (0.98, 1.06)
Middle		1.02 (0.98, 1.06)		1.01 (0.97, 1.05)
Richer		1.02 (0.98, 1.07)		1.01 (0.97, 1.06)
Richest		1.09 (1.04, 1.13)		1.07 (1.02, 1.12)^*^
**Media exposure**				
No		1		1
Yes		1.16 (1.12, 1.19)		1.11 (1.08, 1.15)^**^
**Maternal working status**				
No		1		1
Yes		1.07 (1.04, 1.10)		1.08 (1.06, 1.11)^*^
**Had ANC visit**				
No		1		1
Yes		1.30 (1.21, 1.40)		1.25 (1.17, 1.35)^*^
**Place of delivery**				
Home		1		1
Health facility		1.10 (1.07, 1.14)		1.10 (1.07, 1.14)^*^
**Residence**				
Urban			1	1
Rural			0.90 (0.87, 0.92)	0.99 (0.96, 1.03)
**Sub-Saharan African region**				
East Africa			1	1
Central Africa			0.83 (0.79, 0.86)	0.87 (0.84, 0.91)^*^
Southern Africa			1.04 (0.99, 1.09)	1.02 (0.97, 1.07)
West Africa			0.98 (0.95, 1.01)	1.04 (1.01, 1.08)*
**Distance to the health facility**				
Not a big problem			1	1
Big problem			0.94 (0.91, 0.97)	0.95 (0.93, 0.98)^*^
**Community media exposure**				
Low			1	1
High			1.07 (1.04, 1.10)	1.04 (1.02, 1.07)^*^
**Community maternal education**				
Low			1	1
High			1.04 (1.01, 1.08)	1.02 (0.99, 1.06)
**Community poverty**				
Low			1	1
High			0.99 (0.96, 1.02)	1.01 (0.98, 1.03)
**Model comparison**				
LLR	-15,669.87	-15,533.28	-15,599.73	-15,503.38
Deviance	31,339.74	31,066.56	31,199.46	31,006,76
AIC	31,341.74	31,094.55	31,217.46	31,050.77
BIC	31,349.47	31,202.79	31,287.04	31,220.85

*AIC* Akaike Information Criteria, *ANC* Antenatal Care, *BIC* Bayesian Information Criteria, *LLR* Log-likelihood Ratio, *PR* Prevalence Ratio

* *p*-value < 0.05, ** *p*-value < 0.01

## Discussion

The study found that the prevalence of mothers’ healthcare-seeking behavior for symptoms of ARIs among children under age five in SSA was 64.9% with considerable variation across countries. This finding is higher than the previous study reported in the Philippines (53.4%) [43]. Improved access to healthcare and a better understanding among mothers of the severity of acute respiratory infections in children under five might be the reason for the increasing proportion of children under five seeking medical attention for symptoms of ARIs [44]. In addition, in sub-Saharan African countries to tackle the problem of access to health care and financial crisis, community health insurance has been established and is currently widely implemented to enhance communities' healthcare-seeking behavior for illnesses [45]. However, this finding was lower than studies reported in high-income countries [20, 46, 47], this could be due to the scarcity of public financing, a poor public system in the supply of essential health care, and the government's inability to allocate adequate financing to its health system in sub-Saharan African countries compared to the developed nations [48, 49].

Consistent with studies reported in Ghana [50], Bangladesh [19], and Nepal [51], maternal education was found significant predictor of health-seeking behavior for symptoms of ARIs among under-five children. This could be due to educated mothers having good access to information on common childhood infections like acute respiratory infections and their symptoms [52, 53]. Besides, education can empower women to make health care decisions and their ability to seek health care for their children [54]. Another significant predictor was household wealth status. This is in line with a study's findings in Indonesia [55] and SSA [14]. This could be attributable to mothers with good incomes who can pay for medical care and afford medical assurance hence they are capable of seeking health care whenever their child feels sick [26].

Media exposure enhances mothers' healthcare-seeking behavior for symptoms of ARIs among under-five children. This is in line with a study reported in rural Bangladesh [56], this could be due to media exposure enables mothers to seek key messages on upper respiratory infection symptoms, its severity, and management, which in turn, enhances mothers' seek care at the health facility [57]. Media exposure is a vital tool for health promotion and has a significant positive impact on healthcare service utilization [58]. Health facility delivery and ANC visits found significant positive predictors of the healthcare-seeking behavior of mothers for symptoms of ARI among children under age five. This is supported by previous study findings [28, 55], this is because having previous exposure to maternal and child health care services is important to their awareness and trust about health care services, the relevance of seeking health care services, and the accessibility of services sought for the treatment of their children. Having ANC visits and giving birth at a health facility enhances their interest in health care services including health care providers and strongly motivated to seek health care when their child shows signs and symptoms of diseases [59, 60].

Another important predictor significantly associated with the mother’s healthcare-seeking behavior was the distance to a health facility. Mothers who had big problems with health facilities had a decreased chance of seeking healthcare for symptoms of ARI among children under five compared to mothers where the distance to health facilities was not a big problem. It is in line with previous studies [50, 61], this might be because mothers who with a big problem reaching a health facility need to use transportation facilities or travel a long distance to reach the facility, which in turn contributes not to seeking health care for their child [62]. In addition, mothers who are near the health facility are more likely to visit health care facilities as they are free from transportation costs to reach the health facility [13, 63, 64]. Compared to mothers who had no work, working mothers had an increased chance of seeking health care for symptoms of ARIs among under-five children. This finding is consistent with studies reported in Nigeria [21], and India [65], this is because women who are working have relatively higher incomes, and contact with different individuals, this in turn could increase women’s economic independence, access to information and strengthen their autonomy in making health care decisions for their children [66]. The prevalence of seeking healthcare for symptoms of ARIs of children under age five among mothers from the community with high media exposure was higher compared to those who were from the community with low media exposure. This was supported by studies reported in India [65] and West Bengal [67], the possible reason is that health information may improve health-seeking habits through various electronic and print media, such as information about what services are available, where and when to get them, as well as the benefits and risks of accessing specific services.

## Strengths and limitations of the study

The study was done based on the weighted Demographic and Health Survey (DHS) data of 36 SSA to ensure representativeness and to obtain reliable estimates. As the study was cross-sectional, we are unable to show a temporal relationship; however, multilevel modeling was employed to consider the clustering effect to obtain reliable estimates and standard errors. Additionally, our multilevel robust Poisson regression corrects the problem of overestimation of effects size produced by conventional multilevel binary logistic regression model employed in cross-sectional studies and increases the precision of the findings. Moreover, all information related to ARIs and healthcare-seeking behavior was provided by mothers and was not validated by applying medical examinations/investigation, and was thus subjective. Therefore, it is prone to recall bias. Furthermore, important variables such as perceived severity, previous experience of similar illness, and previous history of under-five death were not considered in this study as these variables were not available in DHS. The other limitation was the DHS data for all countries were not collected at the same time and the result could mask the changes.

## Conclusion

This study found that the prevalence of mothers who sought health care for symptoms of ARIs of children under age five was relatively low in SSA compared to the developed nations. The multilevel analysis found that maternal education, maternal occupation, distance to the health facility, sub-Saharan African region, ANC visits, place of delivery, household wealth status, media exposure, and community media exposure as significant factors associated with a mother's healthcare-seeking behavior for symptoms of ARIs of children under age five. These findings highlighted that public health interventions aimed at enhancing maternal education, media access, and maternal health service utilization need to be implemented to promote mothers' healthcare-seeking behaviour for symptoms of ARIs among children under age five.

## Data Availability

Data is available online and you can access it from www.measuredhs.com.

## Abbreviations

APR: Adjusted Prevalence Ratio
ARIs: Acute Respiratory Infection symptoms
CI: Confidence Interval
DHS: Demographic and Health Survey
ICC: Intra-class Correlation Coefficient
LLR: Log-likelihood Ratio
LMICs: Low-and Middle-income Countries
LR: Likelihood Ratio, MOR; Median Odds Ratio
SSA: Sub-Saharan Africa
WHO: World Health Organization
